# Association between antenatal ultrasound findings and neonatal outcomes in rural Uganda: a secondary analysis

**DOI:** 10.1186/s12884-021-04204-7

**Published:** 2021-11-08

**Authors:** Delia Horn, Erika Edwards, Renny Ssembatya, Kristen DeStigter, Anne Dougherty, Danielle Ehret

**Affiliations:** 1 Pediatrics, The Larner College of Medicine at the University of Vermont, 89 Beaumont Avenue, Burlington, VT 05405 USA; 2 The Larner College of Medicine at the University of Vermont, 89 Beaumont Avenue, Burlington, VT 05405 USA; 3 Imaging the World Africa, Plot 435, Naalya-Namugongo Road, Kampala, Uganda; 4 Radiology, The Larner College of Medicine at the University of Vermont, 89 Beaumont Avenue, Burlington, VT 05405 USA; 5 Obstetrics and Gynecology, The Larner College of Medicine at the University of Vermont, 89 Beaumont Avenue, Burlington, VT 05405 USA; 6 Pediatrics, The Larner College of Medicine at the University of Vermont, 89 Beaumont Avenue, Burlington, VT 05405 USA

**Keywords:** Ultrasound, Neonatal, Perinatal, Mortality, LMIC, LIC, Stillbirth, Resuscitation

## Abstract

**Background:**

Although the use of prenatal ultrasound services has increased in low- income and lower middle-income countries, there has not been a concurrent improvement in perinatal mortality. It remains unknown whether individual ultrasound findings in this setting are associated with neonatal death or the need for resuscitation at delivery. If associations are identified by ultrasound, they could be used to inform the birth attendant and counsel the family regarding risk, potentially altering delivery preparedness in order to reduce neonatal mortality.

**Methods:**

This was a secondary analysis of data collected from a prospective cohort. Data was gathered at Nawanyago Health Centre III in Kamuli District, Uganda. Participants included pregnant women who received second and third trimester prenatal ultrasound scans and delivered at that center between July 2010 and August 2018. All ultrasounds were performed at Nawanyago and deliveries were attended solely by midwives or nurses. Predictor variables included the following ultrasound findings: fetal number, fetal presentation, and amniotic fluid volume. The primary outcome was bag-mask ventilation (BMV) of the neonate at delivery. The secondary outcome was stillbirth or neonatal death in the delivery room.

**Results:**

Primary outcome data was available for 1105 infants and secondary outcome data was available for 1098 infants. A total of 33 infants received BMV at delivery. The odds of receiving BMV at delivery was significantly increased if amniotic fluid volume was abnormal (OR 4.2, CI 1.2-14.9) and there were increased odds for multiple gestation (OR 1.9, CI 0.7-5.4) and for non-vertex fetal presentation (OR 1.4, CI 0.6-3.2) that were not statistically significant. Stillbirth or neonatal death in the delivery room was diagnosed for 20 infants. Multiple gestation (OR 4.7, CI 1.6-14.2) and abnormal amniotic fluid volume (OR 4.8, CI 1.0-22.1) increased the odds of stillbirth or neonatal death in the delivery room, though only multiple gestation was statistically significant.

**Conclusion:**

Common findings that are easily identifiable on ultrasound in low- and lower middle-income countries are associated with adverse perinatal outcomes. Education could lead to improved delivery preparedness, with the potential to reduce perinatal mortality. This was a preliminary study; larger prospective studies are needed to confirm these findings.

## Background

Neonatal mortality remains high in low-income and lower middle-income countries (LMICs). According to the World Health Organization, 5.3 million children under the age of 5 died in 2015, and the risk of dying is highest in the neonatal period, particularly on the day of birth [1]. The Global Burden of Disease data show that neonatal disorders have risen from the second to the leading cause of death in children under age 5 in Uganda, and are expected to remain the primary cause of death until at least 2040 [2]. Children in sub-Saharan Africa are greater than 16 times more likely to die before age 5 than children in high income countries [1]. Intrapartum-related complications are one of the largest causes of neonatal death [1]. Additionally, 98% of stillbirths occur in LMICs, with an incidence of 28.7/1000 in Sub-Saharan Africa compared with the global average of 18.4/1000 [3]. Ultrasound (US) is currently used as a tool to aid in the screening, diagnosis and treatment of pregnant women in some LMICs. In fact, the World Health Organization recommends at least one ultrasound during pregnancy, yet a large gap remains in access to diagnostic imaging [4]. The exact prevalence of routine prenatal US in antenatal care in LMICs is unknown. In high income countries, detailed US scans reviewed by specialty trained radiologists and obstetricians are routinely used to guide prenatal care and delivery planning. The US services provided in LMICs tend to be vastly different from those available in high income countries. Although potentially more cost effective and suitable for low resource areas, available machines (often hand-held portable) may be less sophisticated for obstetric assessment; task-shifting is common due to the lack of skilled workforce, and the staff performing the scans often have limited training with the ability to capture only basic obstetric findings; additionally, the images are regularly interpreted by these same providers at the point-of-care [5–12].

The use of prenatal US in the LMIC setting has already yielded some benefits, including increased maternal prenatal care [8, 13–15] and increased facility-based delivery [8, 13, 15], as well as improved diagnostic accuracy with regards to confirming pregnancy and gestational age, number of fetuses, placental location, and non-vertex presentation [16–22]. Further, several studies have demonstrated that mid-level providers equipped with some basic training can reliably detect certain basic prenatal ultrasound findings such as fetal growth estimates, fetal presentation, and presence of a multiple gestation [18, 22–25]. However, an association between prenatal US use and improved neonatal outcomes in the LMIC setting has not yet been demonstrated [5, 26–29]. Particular basic prenatal US findings might be associated with neonatal outcomes, including the need for resuscitation or death at delivery. If such associations were identified, they could be used to inform the birth attendant and counsel the family regarding risk, potentially altering the location of delivery and preparedness of the team responsible for the neonate. This could lead to new algorithms of care that could improve neonatal outcomes and reduce neonatal mortality. Therefore, there is an urgent need to establish which US findings in LMICs are associated with the status of the neonate at delivery. In the absence of such knowledge the use of antepartum US in the resource limited setting cannot be optimized to improve neonatal outcomes.

Current evidence supports the hypothesis that an association between basic prenatal US findings and neonatal outcomes in LMICs exists. It is well known that certain prenatal factors are associated with poor neonatal outcomes at delivery. For example, breech presentation, especially when delivered vaginally, is associated with increased morbidity and mortality when compared to vertex presentation [30]. Multiple gestation is associated with an increased risk of several factors, including discordant growth, gestational diabetes, and preterm birth, all of which can adversely affect the neonate [31]. Polyhydramnios is associated with a high risk of poor pregnancy outcomes [32, 33]. These conditions are known to place the neonate at risk; however, in high-income countries these risks are mitigated by interventions, including ultrasound, in the prenatal and neonatal period. Similar interventions are not readily available in LMICs, putting these infants at even greater risk for poor outcomes. Geographic remoteness in LMICs, and the need for time to gather funds necessary to travel to a clinic or hospital, further contributes to the need for early detection so that safe deliveries can be planned. The goal of this study is to determine which US findings are most strongly associated with the need for neonatal resuscitation or death at delivery in the LMIC setting, so that this information can be used to optimize neonatal health.

## Methods

### Study design

This is a secondary analysis of data collected under the prospective cohort study “Evaluation of a Low-Cost Ultrasound Scan Protocol for Reducing Maternal and Perinatal Morbidity and Mortality in Ultrasound-naïve Rural Communities in Uganda”. Results from that data have previously been reported [13, 14]. The purpose of our current study was to determine if an association between prenatal US findings identified in a rural low-income country (LIC) and the status of the neonate at delivery could be identified. We partnered with Imaging the World (ITW), a non-profit organization that provides ultrasound technology and ultrasound training in LMICs in order to conduct this study.

### Study population

Mothers who had at least one prenatal US performed at Nawanyago Health Center III (HCIII) in Kamuli District, Uganda and also delivered at that center were included in the study, along with their infants. ITW has been active at Nawanyago HCIII since 2010. Mothers were excluded if they delivered elsewhere, had no prenatal US scan, or if outcome data was not recorded. Between July 2010 and August 2018 data were collected on 6183 mothers at Nawanyago HCIII. Of these, all existing data were entered into REDCap for 1064 mothers who delivered at that center by August 2018, and their 1112 infants. The other 5119 mothers did not have any outcome data available; either the data were not collected or the mothers delivered somewhere other than Nawanyago HCIII. All mothers and infants were black African. US was performed when the mother first presented for care, and again at 32 weeks if possible, though later if she presented to care later. Thirty-two weeks was chosen for a number of reasons related to cultural context: at the start of the study, mothers often presented for their only prenatal care around 32 weeks due to local custom; a scan at 32 weeks often provided more useful information for delivery planning and was easier for a minimally-trained sonographer to interpret than a scan at term or during labor; and at 32 weeks gravid women were often still able to walk the potentially long distance to the clinic if they lived in a remote area. Finally, multiple gestation is very common in this population and so an early 3^rd^ trimester scan was deemed preferable as these mothers often deliver early. If the mother presented late to prenatal care, which was common, she may have only received one scan. Several mothers only presented in labor and were only scanned immediately prior to delivery. It is important to note that if the scan was done at or prior to 32 weeks, the fetal lie at delivery could have varied considerably from what was recorded at the time of the US, but the scan informed the delivery planning. Approximately 80% of women coming to Nawanyago HCIII for prenatal care during the study time period received an US scan.

### Setting

Health Centre III facilities in Uganda are generally staffed by mid-level providers, including clinical officers and midwives, without physicians on site. Nawanyago HCIII along with Bupadhengo HCIII, a government facility 4 kilometers (km) away, serve the 23,000 people who live in the rural Nawanyago sub-district of Kamuli District. HCIII deliveries are exclusively attended by midwives or nurses. Cesarean delivery is not provided. Nawanyago HCIII refers to Kamuli Mission Hospital, a large referral hospital about 30 minutes away by motor vehicle. Critically ill patients can be transported by the HCIII ambulance when it is running and there is a driver available.

Data were collected on consenting patients at Nawanyago HCIII under the original study. All US findings were obtained using a Philips ClearVue 350 US machine with a C5-2 transducer, provided by ITW. Scans were performed by nurses or midwives trained by ITW using a sweeps protocol specifically designed for the LMIC setting [34, 35], and were sent for remote interpretation by expert sonographers at Kamuli Mission Hospital.

### Data collection

Informed consent for data collection was obtained prior to study entry for all participants. Data were collected by trained staff. Data were recorded in hardcopy logbooks at Nawanyago Health Center III and stored in a locked cupboard inside a locked room with security monitoring. Data were de-identified and labeled with a study ID that was unique per pregnancy before being entered into a REDCap database or otherwise sent off site. Identifiable patient health information and the study ID only occurred together in the secured logbooks mentioned above, which were accessed only by the HCIII Director and the Imaging the World Africa (ITWA) Data Lead. For this current study, all de-identified data from the logbooks were entered into REDCap by the ITWA Data Lead for further analysis.

### Outcomes

Based on completeness of maternal records and frequency of pathologies, the predictor variables eligible for inclusion in our analysis were fetal presentation, amniotic fluid volume, and fetal number. We intended to look at placental location as a predictor variable as well, but abnormal placental location (placenta previa) was not seen with enough frequency to be analyzed in this cohort. Fetal presentation was defined as vertex or other (any non-vertex position), amniotic fluid volume was defined as normal (Amniotic Fluid Index 8 cm – 18 cm [36, 37]) or other (an Amniotic Fluid Index either above or below these limits), and fetal number was defined as singleton or multiple gestation. The primary outcome was receipt of bag-mask ventilation (BMV) at delivery. The secondary outcomes were the composite of death in the delivery room or stillbirth, and Apgar scores at 1 and 5 minutes.

We compared infants with and without the primary outcome with regards to the following variables to assess the similarity of the groups (Table 1): estimated gestational age at delivery (term (≥37 weeks gestation) or preterm (<37 weeks gestation)); neonatal birth weight; neonatal sex; and mode of delivery (vaginal or breech vaginal). Post-hoc, we included maternal age and maternal gravidity.

**Table 1:** Population characteristics

Characteristic	Bag Mask Ventilation *N*=33	No Bag Mask Ventilation *N*=1072
Maternal characteristics		
Mode of delivery, n (%)		
Vaginal	25 (76)	1050 (98)
Breech Vaginal	8 (24)	22 (2)
Missing/unknown	0	0
Gravidity, n (%)^a^		
Primigravida	15 (45)	218 (20)
Multigravida	8 (24)	438 (41)
Grand multigravida	9 (27)	327 (31)
Missing/unknown	1	89
Maternal Age in years, mean (SD)^b^	25.3 (6.5)	25.9 (5.9)
Infant characteristics		
Estimated gestational age, n (%)		
Term	28 (85)	1041 (97)
Pre-term	5 (15)	31 (3)
Missing/unknown	0	0
Birth Weight in grams, mean (SD)^c^	2.952 (0.890)	3.301 (0.544)
Sex, n (%)^d^		
M	19 (58)	499 (47)
F	12 (36)	533 (50)
Missing/unknown	2	40

^a^*N*=1022

^b^*N*=861

^c^*N*=1104

^d^*N*=1063

### Data analysis

After entry into REDCap, data were analyzed using STATA 15.1. Logistic regressions were performed to examine the relationships between our predictor variables and outcome variables. Separate regression models were performed to look at the effect of gravidity (primigravida, multigravida, grand multigravida) on our primary outcome. A *p* value <0.05 was considered statistically significant.

We performed a bivariate logistic regression by gravidity and looked at gravidity as a potential effect modifier. The three sub-groups that were analyzed in this multivariate logistic regression were primigravid (first pregnancy), multigravida (2-4 pregnancies) or grand multigravida (≥5 pregnancies).

The study was approved by the Mengo Hospital Research and Ethics Committee at Mengo Hospital in Uganda and the Uganda National Council for Science and Technology, and also received a “Not Human Subjects” exemption from the Research Protections Office at the University of Vermont in the United States. As this was a secondary analysis of de-identified data, informed consent was waived by the following ethics committees: the Mengo Hospital Research and Ethics Committee, the Uganda National Council for Science and Technology, and the Research Protections Office at the University of Vermont. All methods were carried out in accordance with relevant guidelines and regulations.

## Results

A higher proportion of those who received BMV were delivered via breech vaginal delivery, had primigravid mothers, and were born preterm (Table 1).

There was no meaningful difference in mean maternal age, infant sex or birth weight between the two groups.

Overall, 1105 of the 1112 infants had primary outcome data available (Table 2).

**Table 2:** Primary outcome unadjusted results

	Bag mask ventilation (*N*=33)	No bag mask ventilation (*N*=1072)	Odds ratio for bag mask ventilation use
No. Fetuses, n (%)^a^			
Singleton	28 (85)	957 (89)	1.9 (0.7, 5.4)
Multiples	5 (15)	80 (7)	1.0
Missing/unknown^d^	0	35	
Fetal presentation, n (%)^b^			
Vertex	24 (73)	835 (78)	1.4 (0.6, 3.2)
Other	9 (27)	200 (19)	1.0
Missing/unknown^d^	0	37	
Amniotic fluid, n (%)^c^			
Normal	30 (91)	1012 (94)	4.2 (1.2, 14.9)
Abnormal	3 (9)	24 (2)	1.0
Missing/unknown^d^	0	36	

^a^*N*=1070

^b^*N*=1068

^c^*N*=1069

^d^Dropped for adjusted analyses

The seven infants who did not have primary outcome data were all the second infant delivered in a set of twins. In total, 33 infants received BMV at delivery. All predictor variable data was available for the 33 infants who received BMV and for infants diagnosed with the composite secondary outcome of stillbirth or neonatal death in the delivery room; however, there was missing data related to each predictor variable for infants who did not have the primary or secondary outcome of interest. The odds of receiving BMV at delivery was significantly increased if amniotic fluid volume was abnormal on prenatal US (OR 4.2, CI 1.2-14.9) and increased odds for multiple gestations (OR 1.9, CI 0.7-5.4) and for fetal presentation other than vertex on US (OR 1.4, CI 0.6-3.2, Table 4) that were not statistically significant. Both oligohydramnios and polyhydramnios were associated with the need for BMV. Given the relative infrequent nature of either finding, statistical power in comparing the two descriptions of abnormal fluid volumes is limited.

Twenty infants were stillborn or died in the delivery room, with 12 stillbirths and 8 neonatal deaths (Table 3).

**Table 3:** Secondary outcome unadjusted results

	Stillborn/Neonatal death (*N*=20)	Alive (*N*=1078)	Odds ratio for stillborn/Neonatal death
No. Fetuses, n (%)^a^			
Singleton	15 (75)	969 (90)	4.7 (1.6, 14.2)
Multiples	5 (25)	78 (7)	1.0
Missing/unknown^d^	0	31	
Fetal presentation, n (%)^b^			
Vertex	16 (80)	841 (78)	0.7 (0.2, 2.3)
Other	4 (20)	204 (19)	1.0
Missing/unknown^d^	0	33	
Amniotic fluid, n (%)^c^			
Normal	18 (90)	1021 (95)	4.8 (1.0, 22.1)
Abnormal	2 (10)	25 (2)	1.0
Missing/unknown^d^	0	32	

^a^*N*=1067

^b^*N*=1065

^c^*N*=1066

^d^Dropped for adjusted analyses

In an analysis of the 1098 infants with secondary outcome data available, both multiple gestation (OR 4.7, CI 1.6-14.2) and abnormal amniotic fluid volume (OR 4.8, CI 1.0-22.1) increased the odds of stillbirth or neonatal death in the delivery room (Table 4), though only multiple gestation was statistically significant.

**Table 4:** Adjusted primary and secondary outcomes

	Bag mask ventilation Odds ratio (95% CI)	*P*-Value	Stillborn/Neonatal death Odds ratio (95% CI)	*P*-Value
No. Fetuses		0.21		0.006
Multiples Singleton	1.9 (0.7, 5.4) 1.0		4.7 (1.6, 14.2) 1.0	
Fetal Presentation		0.413		0.574
Other Vertex	1.4 (0.6, 3.2) 1.0		0.7 (0.2, 2.3) 1.0	
Amniotic fluid		0.025		0.045
Abnormal Normal	4.2 (1.2, 14.9) 1.0		4.8 (1.0, 22.1) 1.0	

This fit with the primary outcome results showing that these infants were more likely to receive BMV at delivery. In this secondary analysis fetal presentation was not predictive of stillbirth or neonatal death in the delivery room (OR 0.7, CI 0.2-2.3). For infants receiving BMV, the mean Apgar score at 1 minute was 5 and the mean score at 5 minutes was 8. For infants who did not receive BMV, the mean Apgar score at 1 minute was 9 and the mean score at 5 minutes was 10.

We looked at maternal gravidity as a potential effect modifier and found there was no effect modification when stratifying for gravidity (Table 5).

**Table 5:** Adjusted results by gravidity

	Primigravid	*P*-Value	Multigravid	*P*-Value	Grand Multigravid	*P*-Value
	Bag mask ventilation Odds ratio (95% CI)		Bag mask ventilation Odds ratio (95% CI)		Bag mask ventilation Odds ratio (95% CI)	
No. Fetuses						0.009
Multiple					6.8 (1.6-28.7)	
Singleton					1.0	
Fetal presentation		0.461		0.601		0.596
Other	1.6 (0.4 – 6.2)		1.6 (0.3 – 8.8)		1.5 (0.3 – 6.3)	
Vertex	1.0		1.0		1.0	
Amniotic fluid volume						0.000
Abnormal			30.8 (6.2 – 152.3)			
Normal			1.0			

Empty cells represent categories for which the result was not calculable with the available data

Abnormal amniotic fluid volume in multigravid mothers (OR 30.8, CI 6.2-152.3) and multiple gestation in grand multigravid mothers (OR 6.8, CI 1.6-28.7) were associated with increased risk for receiving BMV at delivery.

## Discussion

Common findings that are easily identifiable on US in the LIC setting (including abnormal amniotic fluid volume, multiple gestation, and non-vertex fetal presentation) are associated with adverse neonatal outcomes. This knowledge can be used by care providers in LICs to guide delivery preparedness and improve outcomes for neonates around the time of birth.

These findings are consistent with what has been demonstrated in high-income countries. Previous studies conducted in high-income countries have demonstrated that breech presentation is associated with increased morbidity and mortality when compared to vertex presentation [30]. Multiple gestation is associated with an increased risk of several factors which can adversely affect the neonate, including preterm birth and growth restriction [31]. Abnormal amniotic fluid volume has been shown to be associated with a high risk of poor pregnancy outcomes [32, 33]. The results of our study suggest that these associations are consistent in a LIC. Of note, the dating of our ultrasounds varied widely as many mothers only presented once to care. Many mothers only received their ultrasound scan on the day of delivery, while others had their only scan performed weeks to months before delivery. If the scan was done at or prior to 32 weeks, the fetal lie at delivery could have varied from what was recorded at the time of the US. Only 30 babies were delivered breech, while 209 were non-vertex on ultrasound.

This study demonstrates that high-risk ultrasound findings, which are identifiable in the LIC, are associated with the status of the neonate at delivery. Educating providers about these associations could improve delivery preparedness leading to improved outcomes for neonates. If HCIII health care workers discover these high-risk findings on US, they could use this knowledge to help guide the family to seek an appropriate level of care at the time of delivery. Further, if this information is available to the delivering clinician when the mother presents in labor, it could allow that clinician to anticipate a potentially depressed neonate at delivery so they could better prepare for this outcome with appropriate equipment and personnel. This knowledge has the potential to have the greatest impact if coupled with resuscitation training and appropriate resource allocation. Currently, there is a shortage of nurses and midwives competent to perform neonatal resuscitation in Uganda, however there is an ongoing national effort to provide this training to health workers who perform deliveries. It would be timely to couple this national effort with an increased ability to predict neonatal status at delivery.

Interestingly, in this study there was no effect modification when controlling for gravidity. This is important given that gravidity in Uganda tends to be very high, even for a LIC. Cultural practices lead to grand multigravida in a large percentage of women (per the 2016 Uganda Demographic and Health Survey [38], women in Uganda have an average of 5.4 children, and in rural areas this increases to an average of 5.9 children per woman). We did not see that our predictor variables were more strongly associated with poor neonatal outcomes in multigravida or grand multigravida women. However, this study was not adequately powered to look at effect modification by gravidity, and so larger studies are needed.

This secondary analysis was limited by gaps in data collection and data quality. The primary outcome of BMV occurred in a surprisingly small number of cases. Existing data suggests that roughly 10% of infants will require BMV at delivery, which would have been about 110 infants from a sample this size [39]. When the authors visited Nawanyago HCIII at the conclusion of the study, health workers there reported 1-2 infants requiring BMV per month, which would have yielded 120-240 infants with the primary outcome of interest over this 10 year period (as opposed to 33). However, the data were internally consistent. A one-minute Apgar of 5 or less was only seen in the infants who were documented as having received BMV. There were 7 infants for whom the primary outcome was not recorded, and all were the second twin in a twin set. There were 6 infants who were recorded as being stillborn who did not receive BMV, but the timing of intrauterine demise for these infants is unclear. Further, the secondary outcome of stillbirth or death in the delivery room also occurred less frequently in this cohort than would be expected based on national numbers. Uganda reported a stillbirth rate of about 1.8-2.1% during this study period [1], and in this cohort only 1% were stillborn. Finally, 15% of the infants requiring BMV were preterm infants. This represents a potential confounder as these infants are more likely to require resuscitation due to their preterm status at delivery. Only 2 out of 5 preterm infants requiring BMV had abnormal findings on US. However, the absolute numbers are too small to determine whether this was a confounding factor, or whether the associations being investigated held true for preterm infants as well as term infants.

Once all data were entered into REDCap and available for analysis, it was noted that data pertaining to many variables were either not collected at the clinic level or not entered into REDCap (including information on maternal and infant demographics and pertinent health history). Additionally, some areas of “complete” data collection were anecdotal outliers, and so were excluded from the study. For example, only a total of 18 out of 1064 mothers were recorded as having malaria at some time in their pregnancy, however positive malaria status was only recorded if definitive malaria testing was done. A mother would only undergo definitive malaria testing if she was hospitalized with her symptoms or if she paid out-of-pocket for a test. Most pregnant women in this endemic region are simply treated if they show symptoms of malaria, without a positive test result being recorded – therefore, this led to a gap in data collection reflecting the prevalence of disease. After data analysis was complete, results were discussed with practitioners at Nawanyago Health Center III. Important feedback included that data collection and data quality assurance were challenging due to chronic understaffing.

The results of this study confirm suspicions that certain high-risk findings, when identified on US in this rural clinic in a low-income country, are associated with the status of the neonate at delivery. This information can be used to help providers in LICs better prepare for these deliveries. However, the study was limited by the available data. We intend to follow this study with capacity building in data collection quality and completeness while simultaneously improving staff and patient preparedness for deliveries when high-risk findings are identified on US. This approach will allow for larger prospective studies to validate the strength and accuracy of these findings, and build upon this work with the goal of improving outcomes for mothers and neonates.

## Conclusions

Common findings that are easily identifiable on US in the LIC setting (including abnormal amniotic fluid volume, multiple gestation, and non-vertex fetal presentation) are associated with adverse neonatal outcomes. This knowledge can be used by care providers in LICs to guide delivery preparedness and improve outcomes for neonates around the time of birth.

## Data Availability

The data that support the findings of this study are available from Imaging the World Africa but restrictions apply to the availability of these data, which were used with permission for the current study, and so are not publicly available. Data are however available from the authors upon reasonable request and with permission of Imaging the World Africa. Please contact Kristen DeStigter with requests for data.

## Abbreviations

BMV: Bag-mask ventilation
HCIII: Health Centre III
ITW: Imaging the World
ITWA: Imaging the World Africa
Km: Kilometer
LIC: Low income country
LMIC: Low and middle income country
US: Ultrasound
